# Total rRNA-Seq Analysis Gives Insight into Bacterial, Fungal, Protozoal and Archaeal Communities in the Rumen Using an Optimized RNA Isolation Method

**DOI:** 10.3389/fmicb.2017.01814

**Published:** 2017-09-21

**Authors:** Chijioke O. Elekwachi, Zuo Wang, Xiaofeng Wu, Alaa Rabee, Robert J. Forster

**Affiliations:** ^1^Lethbridge Research and Development Centre, Agriculture and Agri-Food Canada, Lethbridge AB, Canada; ^2^University of Chinese Academy of Sciences Beijing, China; ^3^Key Laboratory for Agro-ecological Processes in Subtropical Region, Hunan Research Center of Livestock and Poultry Sciences, South-Central Experimental Station of Animal Nutrition and Feed Science in Ministry of Agriculture, Institute of Subtropical Agriculture, Chinese Academy of Sciences Changsha, China; ^4^Institute of Animal Nutrition, Sichuan Agricultural University Ya’an, China

**Keywords:** rRNA-Seq, RNA isolation, metatranscriptomics, microbial diversity, bioinformatics

## Abstract

Advances in high throughput, next generation sequencing technologies have allowed an in-depth examination of biological environments and phenomena, and are particularly useful for culture-independent microbial community studies. Recently the use of RNA for metatranscriptomic studies has been used to elucidate the role of active microbes in the environment. Extraction of RNA of appropriate quality is critical in these experiments and TRIzol reagent is often used for maintaining stability of RNA molecules during extraction. However, for studies using rumen content there is no consensus on (1) the amount of rumen digesta to use or (2) the amount of TRIzol reagent to be used in RNA extraction procedures. This study evaluated the effect of using various quantities of ground rumen digesta and of TRIzol reagent on the yield and quality of extracted RNA. It also investigated the possibility of using lower masses of solid-phase rumen digesta and lower amounts of TRIzol reagent than is used currently, for extraction of RNA for metatranscriptomic studies. We found that high quality RNA could be isolated from 2 g of ground rumen digesta sample, whilst using 0.6 g of ground matter for RNA extraction and using 3 mL (a 5:1 TRIzol : extraction mass ratio) of TRIzol reagent. This represents a significant savings in the cost of RNA isolation. These lower masses and volumes were then applied in the RNA-Seq analysis of solid-phase rumen samples obtained from 6 Angus X Hereford beef heifers which had been fed a high forage diet (comprised of barley straw in a forage-to-concentrate ratio of 70:30) for 102 days. A bioinformatics analysis pipeline was developed in-house that generated relative abundance values of archaea, protozoa, fungi and bacteria in the rumen and also allowed the extraction of individual rRNA variable regions that could be analyzed in downstream molecular ecology programs. The average relative abundances of rRNA transcripts of archaea, bacteria, protozoa and fungi in our samples were 1.4 ± 0.06, 44.16 ± 1.55, 35.38 ± 1.64, and 16.37 ± 0.65% respectively. This represents the first study to define the relative active contributions of these populations to the rumen ecosystem and is especially important in defining the role of the anaerobic fungi and protozoa.

## Introduction

Current day advances in high throughput next generation sequencing technologies has revolutionized and enhanced the molecular biology and life sciences research landscape. Increases in speed and reductions in cost of sequencing, advances in the development of computational resources and capabilities has resulted in a cost effective ability to study a wide array of biological systems, phenomena and environments at a depth that was hitherto impossible. Culture-independent microbial community studies have particularly benefited from this revolution. Driven primarily by improvements in the ability to study and analyze the well conserved small subunit (SSU) 16S rRNA gene, it is now possible to explore complex heterogeneous communities and ecosystems (in both natural and experimental conditions). This has provided insights into the diversity and functions of microbial life, and the relationships between microbial community dynamics and the physiological states of related biological systems. These technologies have been broadly applied in the study of a wide range of environments and ecosystems. It is becoming more and more usual to find studies which extend beyond elucidating taxonomic profiles, to actually inferring metabolic and functional profiles from such taxonomic distributions and highlighting metabolic and biochemical pathways that might be implicated in such systems (Langille et al., 2013; Aguiar-Pulido et al., 2016; Jiang et al., 2016), thereby advancing the landscape even further.

Many recent microbiome studies have relied on 16S rRNA amplicon sequencing. This approach involves the extraction, amplification and sequencing of a targeted phylogenetic marker region which must (1) be flanked by highly conserved and ‘known’ sequences, (2) be ubiquitous in the required taxonomic region of interest, and (3) be variable enough to discriminate between variant species. This characteristic (of focusing on targeted regions of the genome) makes statistical comparisons and analysis of qualitative/quantitative differential expression information, allele-specific expression measurement and transcript/gene fusion verification, possible. It also allows high throughput, simultaneous measurement of thousands of targets and provides greater specificity and accuracy in the measurement of transcripts of interest (Case et al., 2007; Illumina, 2016a). Sequencing such targeted regions across multiple species in the environment enables easy comparison and analysis of the taxonomic and phylogenetic variations present in the sample set and provides insights into the microbial community composition and dynamics within the environments studied. This technique has been applied in microbial community studies in various environments including soil (Roesch et al., 2007; Lauber et al., 2009; Damon et al., 2012), hydrothermal vents (Sogin et al., 2006) the human microbiome (Grice et al., 2009), rumen microbiome (Kittelmann et al., 2013; Petri et al., 2013a; Snelling et al., 2014) and marine environments (Schauer et al., 2010). There are, however, a number of downsides to amplicon sequencing, the primary one being the possibility of bias due to primer selection (Baker et al., 2003; Hong et al., 2009; Klindworth et al., 2013). In addition, to study different groups of organisms (even in the same environment) an array of primers will have to be used (Kittelmann et al., 2013) and it is then impossible to define their overall relative abundance. It is also susceptible to inadequate knowledge discovery given that it is limited in scope and is unable to discover novel phylotypes, as the design of associated primers is based solely on ‘known’ sequences (Urich et al., 2008; Mao et al., 2012; Ross et al., 2012; Zhou et al., 2015).

Total RNA sequencing (RNA-Seq) on the other hand provides a snapshot of entire transcriptomes. It thus enables measurement of transcript/gene abundances, detection of coding and non-coding RNA, identifying both known and novel features of the transcriptome, and allowing the prediction of potential functions of the collective organisms in a sample (by studying microbiota associated genes), thus leading to a more comprehensive understanding of habitats, ecosystems and biological systems (Fuhrman, 2012; Zhou et al., 2015; Li et al., 2016; Illumina, 2016b). Comparing taxonomic profiles of rumen microbiota using total RNA and targeted amplicon sequencing Li et al. (2016) concluded that RNA-Seq approach showed more diversity and could detect more bacterial and archaeal phylotypes in the rumen. Like amplicon sequencing, RNA-Seq has also been explored for taxonomic assessment of microbial communities in a number of environments including soils (Urich et al., 2008; Mackelprang et al., 2011; Tveit et al., 2014), hydrothermal vents (Lanzen et al., 2011), the human gut (Qin et al., 2012) and the gut of ruminants (Qi et al., 2011; Li et al., 2016).

The analysis of rRNA abundance provides for insights into the activity of microbial cells and their protein synthesis potential (Blazewicz et al., 2013). This is particularly useful in the study of rumen protozoa as it is known that rRNA gene copy numbers in these organisms varies greatly between species and estimates of relative abundance using rDNA based approaches may over estimate or under estimate certain species (Newbold et al., 2015). An evaluation of potential activity using RNA based approaches would provide a more thorough understanding of potential metabolic roles in the highly diverse rumen microbial ecosystem.

Due to the broad coverage of the transcriptome provided by RNA-Seq, the outcomes are not very amenable to comparative statistical analysis beyond descriptive profiles of taxonomy. Supervised or unsupervised community analyses, for example are not usually possible. To address this shortcoming Guo et al. (2015) developed the *SSUsearch* pipeline for metagenomes which aligns shotgun sequence data, and following that extracts reads that mapped to specified regions in the *Escherichia coli* SSU rRNA gene, which are then used for unsupervised clustering and other analyses as would normally be applied to traditional amplicon data. This improvement holds promise for the use of RNA-Seq in phylogeny and taxonomy studies as it can combine the benefits of wider the breadth of coverage (of RNA-Seq) with the specificity, efficiency and flexibility of a targeted (amplicon) approach.

Solid-associated microorganisms make up the predominant proportion of total rumen microbes (McAllister et al., 1994; Forsberg et al., 1997; Yu and Forster, 2005). It is estimated that they account for about 90% of the endoglucanase and xylanase activities in the rumen (Miron et al., 2001). Extracting total RNA from rumen solid-phase samples is essential for experiments aimed at describing the composition or distribution of diversity and exploring metabolic function of rumen microbial community. Wang et al. (2011) reported a method of isolating high-quality total RNA from the solid-phase of ruminal contents based on liquid nitrogen grinding and acid guanidinium-phenol-chloroform (AGPC) extraction (Chomczynski and Sacchi, 1987) followed by column purification. However, as the number of large studies involving 100s or even 1000s of samples is increasing, isolating total RNA could consume substantial amounts of money and time, especially given that the cost of total RNA sequencing is substantially higher than that of the targeted approach. Therefore improvements in methodology that could lead to reductions in expense and efforts while ensuring the integrity and quality of the RNA yield would be helpful.

Maintaining the stability of RNA molecules is critical to the extraction of high quality RNA. One common protocol that assures the stability of total RNA isolated involves the use of TRIzol reagent (Rio et al., 2010). For rumen solid-phase samples this process involves grinding samples of rumen digesta in liquid nitrogen for specified periods and more importantly, adding TRIzol reagent to the mix soon after, before thawing and subsequent extraction. Changes in this method can be introduced at three different points: (1) the mass of initial sample for grinding, (2) the mass of ground sample used in the actual extraction, and (3) the amount of TRIzol reagent used. Current practice in our laboratory usually starts with the collection of a 500 g representative sample from the rumen, subsampling 5 g of solid-phase rumen content, which after grinding is split into 4 microfuge tubes (1.25 g in each) into which 12.5 mL of TRIzol reagent is added – resulting in a 10:1 TRIzol/ground matter proportion. This study compared RNA yield and quality arising from the use of lower masses of solid-phase digesta for the initial grinding and for RNA isolation. It also investigated the possible impacts of using lower amounts of TRIzol per unit weight of ground solid material on RNA yield and quality. Taxonomic compositions of the ruminal microflora of the cattle solid-phase samples where the less expensive (lower mass of starting material, lower TRIzol/ground mass ratio) total RNA extraction approach was applied were then evaluated. A bioinformatics pipeline to analyze total SSU rRNA reads was developed. Finally, using a modified *SSUsearch* pipeline (Guo et al., 2015), RNA-Seq reads mapped to a target V4 region were extracted for statistical analysis and comparisons.

## Materials and Methods

### Animals and Rumen Sampling

The rumen-cannulated cattle used in this study were housed in a tie-stall barn at the Agriculture and Agri-Food Canada Lethbridge Research Centre in Lethbridge, AB, Canada, and were treated in accordance with the guidelines set by the Canadian Council on Animal care (CCAC, 2009). Solid-phase samples were obtained by placing whole ruminal contents in a heavy-walled 250 mL beaker and by separating the particulate and liquid phases using a Bodum coffee filter (Bodum Inc., Triengen, Switzerland). Samples were immediately flash-frozen in liquid nitrogen within 5 min after being withdrawn from the animal, and then transferred to the laboratory and stored at -80°C for further processing.

### Total RNA Isolation and Evaluation of RNA Quantity and Quality

#### Comparison of Different Amounts of Solid-Phase Sample Used for Grinding and RNA Isolation

To test the effects of different amounts of solid-phase sample used for grinding and RNA isolation on RNA quantity and quality, a solid-phase sample was first manually ground into crude powder in liquid nitrogen using a mortar and pestle. 3 and 2 g of the crude powder was respectively ground for an additional 5 min in liquid nitrogen using a Retsch RM200 grinder (Retsch GmbH, Haan, Germany). After that 0.6 and 0.3 g of the resulting fine powder was weighed into 50 mL tubes and mixed with 6 and 3 mL respectively, of TRIzol reagent. The sample was thawed and incubated at room temperature for 5 min, and RNA was subsequently extracted using the AGPC method (Chomczynski and Sacchi, 1987). The air-dried RNA pellet was re-dissolved in 100 μL of nuclease-free water (Qiagen) and RNA was extracted, both in replicates. A MEGAclear kit (Applied Biosystems/Ambion) was used to purify RNA isolated from the solid-phase samples, and the purification procedures were performed in accordance with the manufacturer’s recommendations.

RNA concentration and integrity were determined using an Agilent 2100 bio analyzer (Agilent Technologies, Mississauga, ON, Canada) and RNA 6000 Nano kit (Agilent Technologies) according to the manufacturer’s instructions. Given that prokaryotes account for the majority of RNA in rumen contents (Yu and Forster, 2005) the prokaryotic total RNA nano assay protocol was used. Large subunit/small subunit (LS/SS) rRNA peak area ratios and RNA integrity numbers (RIN) were analyzed for each RNA sample using the 2100 Expert software version B.02.07 (Agilent Technologies).

#### Comparison of Different Dosages of TRIzol Reagent Used for RNA Isolation

First, a solid-phase sample was manually ground into crude powder in liquid nitrogen, and then 2 g of crude powder was weighed and further ground for 5 min employing the method described above. After grinding, 0.6 g of the resulting fine powder was weighed into 50 mL tubes into which 6, 4.5, or 3 mL of TRIzol reagent were added. The sample was ground and processed to extract RNA, all in replicates. RNA concentration and integrity were estimated using the same methods as described above.

#### Taxonomic Variations amongst Cattle and Samples

Following successful total RNA outcomes from using lower grinding masses and lower TRIzol reagent/mass ratios, twelve solid-phase samples (2 g each), withdrawn from 6 Angus × Hereford cross beef heifers were first ground using the above-mentioned method, and then 0.6 g of fine powder from each of them was weighed into a 50-mL tube and mixed with 3 mL of TRIzol reagent. For each sample, total RNA was extracted in duplicates and the RNA concentration and integrity were estimated using the methods described above. The heifers had been fed a high forage diet comprised mainly of barley straw in a forage-to-concentrate ratio of 70:30 for 102 days.

### Total RNA Sequencing

Total RNA (100 ng) was prepared for sequencing using protocols supplied with the Illumina TruSeq RNA Sample Prep Kit-v2, Set A and B (Illumina, San Diego, CA, United States), with the exception that steps for the enrichment of mRNA or PolyA selection were excluded. Total metatranscriptomic libraries were validated using the DNA 1000 kit (Agilent Technologies) and were quantified using the KAPA SYBR Fast qPCR kit for Illumina Technologies (Kapa Biosystems, Boston, MA, United States). Sample libraries were normalized using the qPCR results and 24 samples were multiplexed and paired end (2 × 300bp) sequenced on an Illumina MiSeq using a MiSeq Reagent v3 600 cycle kit (Illumina, San Diego, CA, United States).

### Bioinformatics Analysis

#### Analysis of the RNA-Seq Data

A pipeline developed in house was used to analyze the total rRNA gene sequences to obtain a snapshot of the microbial community profile of the rumen and to highlight any differences in community composition and/or dynamics. Each pair of paired-end data (R1 and R2) was merged using PEAR (Zhang et al., 2014), run on the command line with default parameters. After merging, the *rRNA–HMM* (Huang et al., 2009) tool of the Rapid Analysis of Multiple Metagenomes with a Clustering and Annotation Pipeline (RAMMCAP) (Li, 2009) was used to identify rRNAs and to separate them into taxonomic domains of Archaea (16S,23S), Eukaryotes (18S, 28S) and Bacteria (16S, 23S). This was effected using hidden markov models. For subsequent steps, the 16S and 18S small sub unit (SSU) reads were subsampled using the *fasta-subsample* tool in the MEME 4.10.2 (Bailey et al., 2009) toolkit, to 20000, 10000, and 2000 sequences for bacteria, eukaryote and archaea respectively. Taxonomy binning for eukaryote and archaeal SSU rRNA sequences was accomplished using BLASTn (Altschul et al., 1990). The subsampled sequences were used as query to search against the SILVA SSURef-111 database using an *e*-value of 1e^-5^. For the bacterial SSU sequences the “classify.seqs” command of MOTHUR 1.33.1 (Schloss et al., 2009) was used to bin them into operational taxonomic units (OTUs) using the SSURef -108 gene and a modified SSURef-108b taxonomy databases as reference. Resulting output files were then parsed to extract relevant information.

#### Community Structure Analysis Based on OTU Clustering

To get some broad community level understanding of the microbial populations represented in the sampled animals the *SSUsearch* pipeline of Guo et al. (2015) was employed, with modifications. This pipeline includes and uses purpose-built HMMs and a reference rRNA gene template – both constructed from the SILVA SSU and LSU ref NR databases (see Guo et al., 2015, for details) to identify rRNA genes/sequences. After the *hmmsearch* step (of the pipeline) the resulting sequence reads were aligned to the rRNA reference genes template using MOTHUR (Schloss et al., 2009). Sequences with >50% match to the reference were used subsequently. Community structure examination involved *de novo* clustering of the resulting rRNA sequences. Comparative analysis were made possible by extraction of OTU reads that mapped to a 250-bp variable region 4 (V4), corresponding to positions 477–727 in *Escherichia coli*. From all the samples sequences from this region were extracted and used in subsequent unsupervised clustering analysis. While the Guo et al. (2015) rRNA gene search pipeline (*SSUsearch*) was used *as is* in this study, the unsupervised analysis pipeline was modified slightly. Apart from the de-replication step where RDP McClust was used all other analysis steps (particularly those covering distance matrix creation and de novo clustering) was done using MOTHUR 1.35.1 (Schloss et al., 2009) The resulting biom file was then transferred to QIMME v1.9.0 (Caporaso et al., 2010) for visualizations and further diversity analyses.

Community structure analyses involved a number of stages including: reads preprocessing and assembly, OTU picking, taxonomy assignment and analysis of diversity. The preprocessing stage involved quality checks, adaptor removal/trimming and merging of forward/ reverse paired-end sequence reads. FastQC v0.11.4 (Andrews, 2010), was used to evaluate sequence quality. Trimmomatic version 0.35 (Bolger et al., 2014) running in paired end mode was used for data trimming and adaptor removal, during which Illumina Truseq3-PE adaptors were removed. Options applied included: a maximal allowance of 2 mismatches, removal of 3 leading or trailing low quality bases, a phred score threshold of 30, a 4-bases wide sliding window, cutting at <15 average quality per base and removing reads less than 36 bases in length. Pear vs. 0.9.6 (Zhang et al., 2014) was used to merge corresponding read pairs (forward and reverse) of the sequences, using default options. Merged sequence file were then subsampled as needed using the *fasta-subsample* tool from the MEME 4.10.2 suite (Bailey et al., 2009). For each sample 70,000 reads were run through the pipeline using SILVA 108 reference taxonomy and SILVA rep-set genes databases for reference. Unsupervised clustering of the V4 rRNA was done according to the *SSUsearch* pipeline (Guo et al., 2015) except for the adaptations outlined earlier. A distance cut-off 0.05 was applied for the clustering and for creation of OTU tables. Community diversity estimations were carried out in QIIME and involved calculation of alpha and beta diversity. Alpha diversity indices evaluated were chao1, Shannon, Simpson and observed_otus. Beta diversity analyses primarily involved the creation of principal co-ordinate analysis (pcoa) plots using the Bray-Curtis dissimilarity index.

Specific phylum level diversity analyses were additionally carried out on the data via modifications of the *SSUsearch* pipeline. Rather than use Hidden Markov Models (HMM) shipped with the *SSUsearch* pipeline, specific HMMs obtained from the Rapid Analysis of Multiple Metagenomes with a Clustering and Annotation Pipeline (RAMMCAP) (Li, 2009) were used within the *hmmsearch* component of the (*SSUsearch)* pipeline - to separate reads into archaea, bacteria and eukaryotic rRNA sequences. The resulting sequence files were aligned to the template (as described earlier). Reads which had a > 50% match to the template and which mapped to the specified 150 bp region were extracted for unsupervised clustering and classification. These downstream analyses, involved *de novo* OTU picking (in QIIME) with a 97% similarity threshold, taxonomic assignment of OTUs using the SILVA v119 database, construction of phylogenetic tree using FastTree (Price et al., 2009), alpha and beta diversity analysis as already explained.

All the sequences in the present study were deposited to the sequence read archive (SRA) of the NCBI under the project accession number SRP103550 and reads SRR5435167 - SRR5435190.

## Results and Discussion

### Comparison of Different Amounts of Solid-Phase Sample Used for Grinding and for RNA Isolation

Functional genomics and gene expression studies depend critically on the ability to isolate high quality RNA. An assessment of RNA integrity is therefore an important first step in obtaining any meaningful data for downstream applications and analysis. Two indices of RNA integrity and quality were used for this assessment (1) ratio of the peak areas of the Large Sub Unit (28S/23S) to the Small Sub Unit (18S/16S) rRNAs (LS/SS ratio) and (2) the RNA Integrity Number (RIN). The eukaryotic 28S/18S ratio may reflect unspecific damage to the RNA, including sample mishandling, post-mortem degradation, massive apoptosis or necrosis (Fleige and Pfaffl, 2006). RNA of high integrity (with no significant degradation) is often shown by intact rRNA peaks and a 28S rRNA band that is approximately twice as intense as the 18S rRNA band (Zou et al., 2008). The RIN has a higher level of acceptance as reflection of RNA quality (Schroeder et al., 2006). While Fleige and Pfaffl (2006) recommended a RIN higher than 5 as good total RNA quality, Jahn et al. (2008) recommended a RIN of 7 be adopted as benchmark of good quality total RNA. For this study a RIN number of between 5 and 7 was adopted as benchmark. Our initial experiment evaluated RNA yield and quality for 3 and 2 g initial mass of solid-phase rumen content using 0.6 and 0.3 g mass of ground material for RNA isolation, with TRIzol reagent introduced at 10x the mass used for isolation. **Table 1** outlines the results of the comparison. With an initial mass of 3 g, using 0.6 g and 0.3 g isolation masses resulted in average RNA yield of 21.9 and 195.5 μg RNA/g of rumen solid respectively, average RIN values of 7.3 and 6.9 respectively, and average LS/SS (Large Sub Unit/Small Sub Unit) rRNA ratios of 1.95 and 1.68 respectively. 2 g initial grinding mass with 0.6 g and 0.3 g isolation masses resulted in 17.4 and 123.5 μg RNA/g of rumen solids respectively, 1.85 and 1.55 LS/SS rRNA ratios respectively, and RIN values of 5.0 and 6.0 respectively. Means and standard errors are outlined in the table. On both fronts – of LS/SS ratio and RIN number – these results align well with accepted indices of good RNA quality, even when the least amount of ground material was used in the RNA isolation protocol. A combination of 2 g grinding mass and 0.6 g isolation mass was chosen as the benchmark and was used to evaluate the proportion of TRIzol reagent that might be optimum for total RNA isolation.

**Table 1:** Comparison of RNA yield and quality from rumen solid (RS) with different initial grinding masses and different masses used for RNA isolation.

Grinding mass (g)	Isolation mass (g)	RNA yield (μg RNA/g RS)	LS/SS rRNA ratio	RIN value
3.0	0.6	21.9 ± 16.9	1.95 ± 0.13	7.3 ± 0.2
	0.3	195.5 ± 220.4	1.68 ± 0.21	6.9 ± 0.8
2.0	0.6	17.4 ± 13.5	1.85 ± 0.13	5.0 ± 1.7
	0.3	123.5 ± 153.2	1.55 ± 0.38	6.0 ± 2.0

*^∗^Data represents mean and standard error for at least 3 replicates per experiment.*

### Comparison of Different Dosages of TRIzol Reagent to Be Used in RNA Isolation

The second stage of this study was aimed at a reduction in the amount of TRIzol that can suitably be used in RNA isolation. It compared outcomes (of RNA yield and quality) when smaller amounts of TRIzol reagent per unit weight of ground matter were used in the process. Using an initial mass of 2 g of rumen solid-phase digesta sample and 0.6 g of the ground solid sample we extracted total RNA using 10x, 7.5x and 5x TRIzol reagent per unit mass of ground matter. Average RNA yield and quality arising from these treatments are outlined in **Table 2** and show that yields of 20.0, 34.1, and 36.5 μg RNA/g RS were obtained from 10x, 7.5x, and 5x TRIzol reagent per unit weight of solid material respectively. LS/SS rRNA ratios were 1.85, 1.75, and 1.65 respectively while RIN values obtained were 7.3, 7.25, and 6.55 respectively. As discussed earlier these values are within acceptable limits for good quality RNA, particularly when the computed standard errors are considered. It is worth noting that in this study the average yields observed from using the least amount of TRIzol reagent evaluated (i.e., 5x) was even higher than that obtained from the amount usually applied. It can be deduced from these results that using 2 g of solid rumen digesta for initial grinding, 0.6 g of ground material for RNA extraction and 3 mL of TRIzol reagent is sufficient for extraction of good quality RNA. This is an improvement on the existing benchmark and will result in significant cost savings for future experiments.

**Table 2:** Comparison of RNA yield and quality from rumen solid (RS) with different amount of TRIzol solution.

TRIzol volume	RNA yield (μg RNA/g RS)	23S/16S rRNA ratio	RIN value
10×	20.0 ± 4.0	1.85 ± 0.07	7.3 ± 0.0
7.5×	34.1 ± 31.5	1.75 ± 0.35	7.25 ± 0.2
5×	36.5 ± 33.4	1.65 ± 0.21	6.55 ± 0.2

*^∗^Data represents mean and standard error for at least 3 replicates per experiment.*

### Taxonomic Composition of Rumen Microbiota from Cattle Samples

**FIGURE 1:**
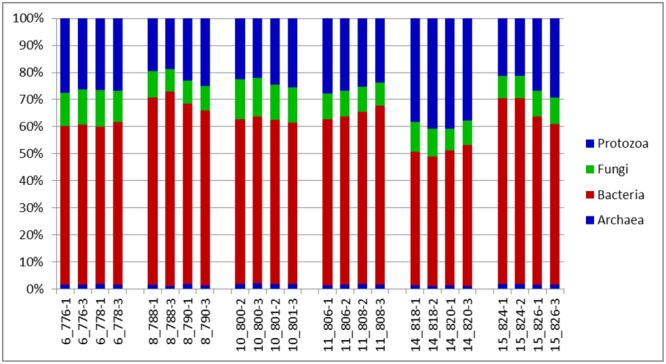
Distributions of phyla (relative abundances), for the total rRNA analyses of solid-phase rumen contents of 6 cattle, 2 samples from each, analyzed in duplicates. The leading numbers 6…15 refer to the animal numbers.

**FIGURE 2:**
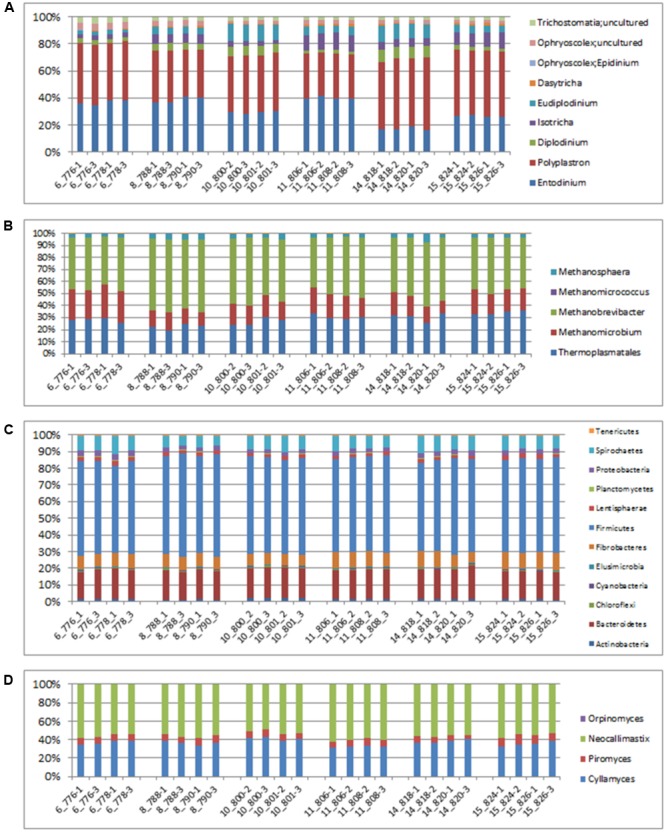
Relative abundances of taxonomic distributions for total rRNA analysis of solid-phase rumen content of 6 cattle showing profiles for **(A)** Protozoa, **(B)** archaea, **(C)** bacteria and **(D)** fungi. The leading numbers (*y* axis) refer to the animal numbers.

An evaluation of taxonomic compositions and community structure profiles resulting from RNA-Seq extracted using the improved benchmark was carried out. This study assessed rumen microbial communities in solid-phase samples obtained from 6 cattle. Two (2) samples were obtained from each animal, total RNA was extracted in duplicate using the improved protocol outlined and the taxonomic composition was then evaluated. In total 11278779 reads were obtained, averaging 469949.125 ± 62638.8 (mean ± SE) reads per sample. Among these 37.01 ± 0.48% and 62.95 ± 0.46% belonged to Small and Large Subunit rRNA respectively. Among the Small Subunit rRNA (SSU rRNA) reads 1.61 ± 0.05, 60.92 ± 1.29 and 37.46 ± 1.31% were archaea, bacteria and eukaryotic SSU rRNA, respectively. Eukaryotic rRNA reads were further broken down into 10.48 ± 0.43 and 26.97 ± 1.28% Fungi and Protozoa rRNA respectively (see Supplementary Table S1). **Figure 1** shows the relative abundances of rRNA transcripts of the microbial phyla represented in the rumen for the cattle sampled. The variation among the animals sampled was clearly seen in these results. The method was also able to produce very reproducible outcomes as the profiles for each of the duplicates for each sample were very similar. Given the reported impact of diet on resulting taxonomic profiles (Chen et al., 2011; Petri et al., 2013a,b; Henderson et al., 2015) the relative active proportions of the various phyla represented in the rumen of cattle sampled in this study appear to be consistent with proportions observed in previous studies involving a high forage diet (Petri et al., 2013a). We also find that this protocol generated similar profiles to that obtained when the usual protocol (5 g initial mass, 1.25 g isolation mass and 10x TRIzol reagent per unit mass of ground mass) was used. **Figures 2A–D** shows profiles of relative active abundances of protozoa, archaea, bacteria and fungi respectively, as observed in the study. An overarching observation across all the investigations is the ability to differentiate animal variation within the sample set, and the ability to evaluate the active contribution of anaerobic fungi and protozoa to the rumen community.

The two most active protozoan genus found in the rumen samples were *Polyplastron* and *Entodinium*, both of which made up an average 74.12% of the protozoan community, with an appreciably higher proportion being found in animal number 14. *Epidinium (Ophryscolex)* only accounted for an average of 0.172% in the rumen samples. The predominant methanogens in the rumen were *Methanobrevibacter, Methanomethylophilus* and *Methanomicrobium*, which made up more than 96% of the active archaea population in the rumen of the cattle sampled, with the *Methanosphaera* making up the remaining. *Methanimicrococcus* was only present in trace amounts. The bacterial community was very similar across all samples analyzed. At the phylum level the largest active group of bacteria in the 24 samples were classified into the phylum *Firmicutes* (57% of the bacteria community) followed by *Bacteriodetes* (17%) *Spirochaetes* (8%) and *Fibrobacter* (7.9%). Other bacteria phyla identified in the rumen which accounted for more than 1% of the active bacteria community were *Lentisphaerae* (2.3%) and *Actinobacteria* (1.7%). At the family level (Supplementary Figure S1) the bacterial community was also found to be similar across all the samples analyzed. These methods were able to distinguish variability in the active fungi community – made up mostly of *Neocallimastix* (55.84 ± 0.61%), *Cyllamyces* (36.54 ± 0.61%) and *Orpinomyces* (7.62 ± 0.27) (**Figure 2D**).

Though the relative active proportions of these microorganisms generally agree with proportions reported in previous studies it is important to note that there are differences. These differences in outcome can be explained by the measurement of the active communities using an RNA approach vs. DNA, the use of a robust isolation technique that enables the isolation of representative communities from fiber samples, and varying experimental conditions including: PCR conditions, choice of primers and cloning vector, other host factors including diets and physiological conditions that differ among experiments (Firkins and Yu, 2006).

### Community Structure Analysis

Extracting reads mapped to a specific region of the 16S rRNA gene enabled deeper comparison and statistical analysis of the data and an assessment of the structure of the microbial community from genomic material obtained using the improved benchmark. It allowed comparative interrogation of the dataset and an analysis of the between sample and within sample diversity contained within the data. A total of 104,467 reads (2.17% of the entire total RNA sequences analyzed) mapped to the specified 250 bp region ranging from 3078 to 9110 reads per sample with a median value of 4146 reads (Supplementary Table S2).

#### Taxonomic Composition

**FIGURE 3:**
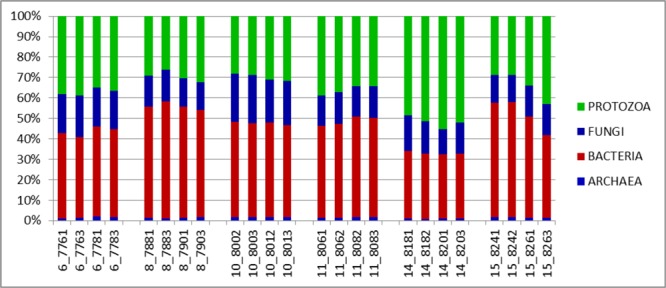
Distributions of phyla (relative abundances), for the V4 region extracted from total rRNA analysis of solid-phase rumen contents of 6 cattle, 2 samples from each, analyzed in duplicates. The leading numbers 6…15 refer to the animal numbers.

The major taxonomic groupings are shown in **Figure 3**. The average active relative abundances of archaea, bacteria, protozoa and fungi were 1.4 ± 0.06, 44.16 ± 1.55, 35.38 ± 1.64, and 16.37 ± 0.65% respectively. Other Eukaryote sub-phyla observed in very small proportions and not included in the broad Fungi and Protozoa groupings included *Alveolata, Diplomonadida, Metazoa, Parabasalia, Rhizaria*, and *Viridiplatae*. A striking observation from **Figure 3** is that it looks similar to the results obtained from the total SSU rRNA-Seq analysis (**Figure 1**), except that the relative active contribution of the phylum bacteria appears to be more pronounced in the rRNA-Seq output. This could also be the result of differences between the *hmmsearch* used in the Guo et al. (2015) pipeline and the *rRNA–HMM* program used in RAMMCAP (Li, 2009) for the total SSU-rRNA analysis. Given that this analysis surveyed only the specified region, whilst the rRNA-Seq analysis assessed the entire SSU rRNA transcriptome within the samples, some differences would be expected. The 18S rRNA transcript of protozoa and fungi is larger than that of the 16S rRNA transcript of bacteria and archaea, therefore an increased number of sequencing reads would be expected for protozoa and fungi in the total SSU rRNA data. However, normalizing the data for SSU length did not significantly change the relative active proportions (data not shown). Although it does appear that the active taxonomic distribution of the isolated region 4 SSU rRNA is slightly different (in proportions) from the entire dataset it provides information that very closely mimics the total SSU rRNA results. Animal variation among the animals sampled was clearly highlighted and therefore this technique will be particularly useful for statistical analysis within experiments of the total active microbial community structure of the rumen.

**FIGURE 4:**
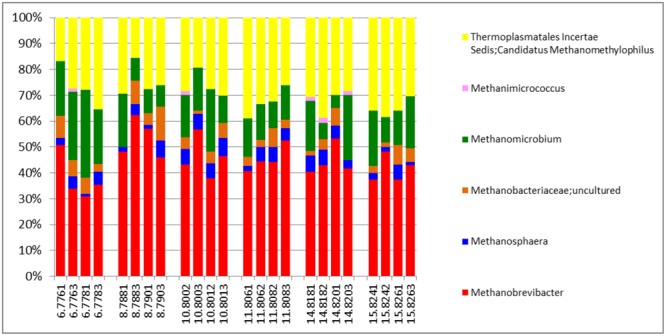
Taxonomic distribution of the archaea community in the V4 region extracted from total rRNA analysis, of solid-phase rumen contents of 6 cattle, 2 samples from each, analyzed in duplicates.

**FIGURE 5:**
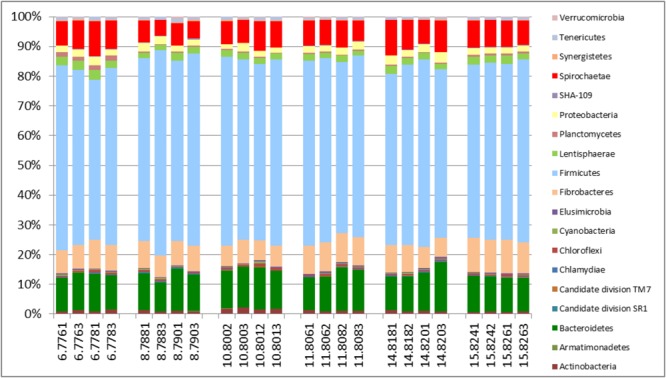
Taxonomic distribution of the bacteria community in the V4 region extracted from total rRNA analysis, of solid-phase rumen contents of 6 cattle, 2 samples from each, analyzed in duplicates.

**FIGURE 6:**
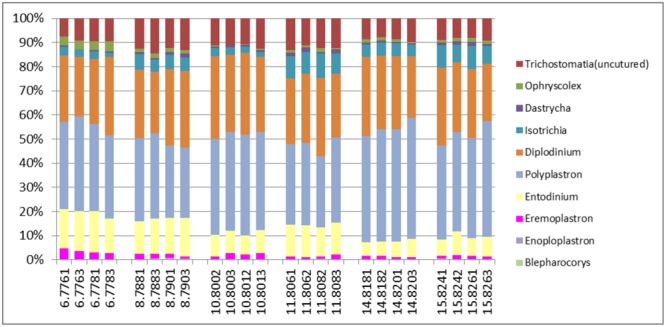
Taxonomic distribution of the Protozoan community in the V4 region extracted from total rRNA analysis, of solid-phase rumen contents of 6 cattle, 2 samples from each, analyzed in duplicates.

The active archaea community in the rumen of the animals sampled (**Figure 4**) were made up predominantly of 3 genera, *Methanobrevibacter, Candidatus Methanomethylophilus* (family: *Thermoplasmatales Incertae sedis*), and *Methanomicrobium* which together make up 90.6% and account for 44.8, 29.6 and 16.2% of the methanogen community respectively. The least contributors to this population were *Methanimicrococcus* which made up 0.3%, *Methanosphaera* which made up 4.3% and an uncultured group of the *Methanobacteriaceae* family which made up 4.7% of the population. This profile is also very similar to that obtained from an assay of the total rRNA (**Figure 2B**). Additionally this targeted approach revealed deeper differentiation and highlighted (though in trace amounts) the presence of two genera, *Methanimicrococcus* and an uncultured *Methanobacteriaceae*, beyond what was possible from the total rRNA assessment. The active bacteria community (**Figure 5** and Supplementary Figures S1–S3) also portrayed a profile similar to that obtained using total rRNA data (**Figure 2C**) with the most predominant subgroups (*Spirochaetes, Proteobacteria, Lentispaerae, Firmicutes, Fibrobacteres*, and *Bacteroidetes*) occurring in similar proportions in both analyses. The active Eukaryota community within the sampled animals is made up primarily of Protozoans (**Figure 6**) and Fungi (Supplementary Figure S4). Ninety eight percent of the fungi community is comprised primarily of members of the family *Neocallimastigaceae*. This is very similar to the outcome from the total rRNA analysis, which differentiated the fungi community into the genera *Neocallimastix, Piromyces* and *Cyllamyces*, all three being members of the family *Neocallimastigaceae*. One major difference observed, however, was that while the total rRNA analysis did not highlight the presence of members of the genus *Orpinomyces* the targeted region show the entire family to be comprised primarily of the genus *Orpinomyces*. An examination of the diversity within the protozoa community also showed a profile in the target region that is markedly different from that observed within the entire total rRNA set. The relative active proportions of the genus *Trichostomatia* was about twice as much in the V4 region than was observed in the rRNA-Seq dataset. The genus *Diplodinium* comprised about three and half times the proportion of the Protozoans in the V4 region than was found in the total rRNA set. For members of the genera *Isotrichia* and *Polyplastron* their average relative active abundances were similar in the two populations while the genus *Entodinium* occurred at two orders of magnitude higher in the total RNA set than was observed in the targeted region. In addition the targeted analysis of the protozoan taxonomic profile highlighted the presence of three other genera – *Eremoplastron, Enoploplastron*, and *Blepharocorys*, all of which occurred in trace amounts.

#### Community Diversity Estimations

**Table 3** shows the alpha diversity calculations observed from the study and presents the mean values (±SE) for indices noted in all the animals sampled (and the replicates). Expectedly species richness (estimated by chao1 index), evenness (estimated by Shannon–Wiener index) and the number of observed OTUs were higher in the entire population sampled than was observed for the individual phyla, as the sample space is much wider. Interestingly, while the observed OTUs were markedly different in all the phyla examined, similarly high value for the inverse Simpson’s index (also estimating evenness and an equal distribution amongst the species represented in the data) were noted for each of the populations examined. This suggests appropriate diversity, for the microbial populations present in the dataset.

**Table 3:** Diversity of the microbial community in rumen solids from 6 cattle examined, evaluated from the V4 region of total rRNA data separated into values for the whole population of microbes in the entire set and values from the different taxonomic phyla examined.

		Phyla (V4)		
	Whole population(V4)^∗^	Archaea	Bacteria	Eukaryote
Chao1	13814.7 ± 242	2418.35 ± 38.5	2129.47 ± 40.4	981.27 ± 25.5
Shannon	10.578 ± 0.05	8.936 ± 0.024	8.439 ± 0.034	6.023 ± 0.058
Simpson	0.996 ± 0.0004	0.994 ± 0.0001	0.989 ± 0.0004	0.952 ± 0.002
Observed OTUs	2740.5 ± 76.75	1018.8 ± 19.7	943.7 ± 18.8	413 ± 12.0
Goods coverage	0.459 ± 0.014	0.728 ± 0.005	0.789 ± 0.004	0.9 ± 0.005

*^∗^Data represents indices for the entire microbial population in the sample set.*

It is notable that with this procedure, for 4 of the 5 alpha diversity indices evaluated, higher values were reported for the archaea phylum even though they only make up about 1.4% of the active microbial population. This gives credence to the capability of extracting useful information about microbial populations using this method. Also, even though there were fewer OTUs observed for the Eukaryotic population than was observed for all the other populations examined the eukaryotic sample appeared to have the highest coverage of all with only 10% of the reads occurring from OTUs that appear only once in the sample. On the other hand, the goods coverage index recorded for the entire population was very low compared to that observed for the specific phyla level analysis. This is due to the fact that only a small subset of the entire population is contained within the V4 region and extracting just this region could have negatively impacted the coverage obtained in the sampling.

**FIGURE 7:**
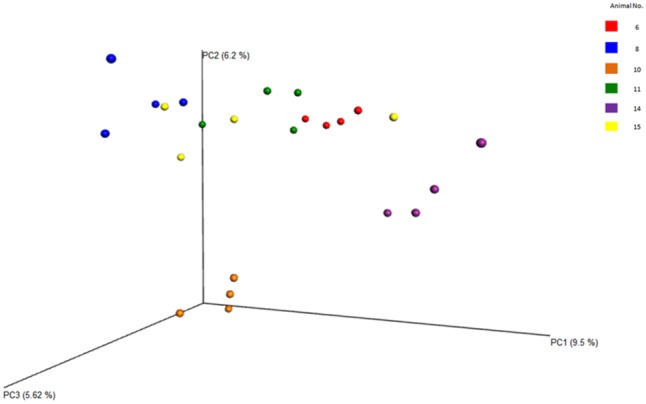
Principal coordinate analysis (PCoA) plots of the diversity among the entire samples and the animals, evaluated from the V4 region. Clearly shows the relatedness/dissimilarities among the samples and among the animals, with the samples from the various animals clustering loosely together.

**FIGURE 8:**
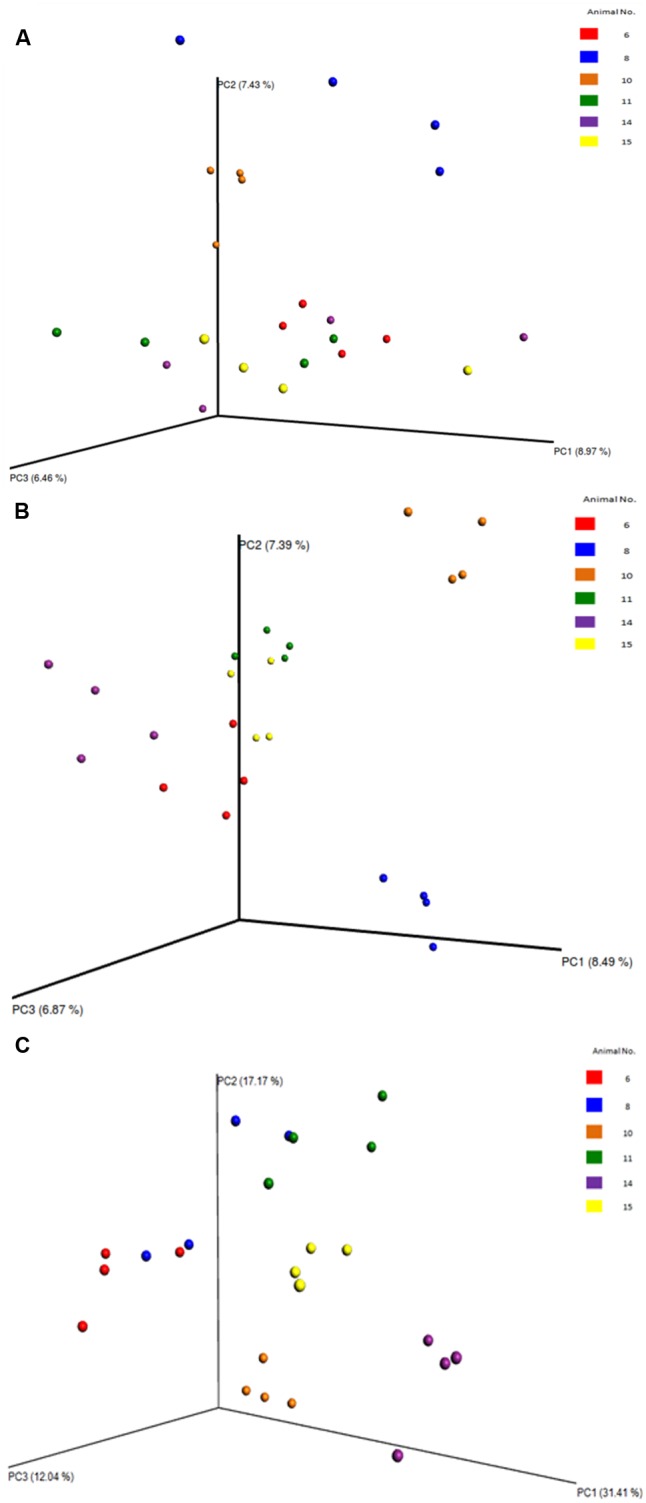
Principal coordinate analysis (PCoA) plots of the diversity among the samples and the animals, evaluated from the V4 region. **(A)** PCoA of the archaea phyla. **(B)** PCoA of the bacterial phyla. **(C)** PCoA of the eukaryotic phyla. Clearly shows the relatedness/dissimilarities among the samples and among the animals, with the samples from the various animals clustering together.

Between sample (beta) diversity of the communities in this study were evaluated using principal coordinate analysis plots with distances calculated using the Bray-Curtis Dissimilarity index. The plots for the entire population (**Figure 7**) and for the specific phyla (**Figure 8**) show the capability of this approach to delineate animal level variation with clarity – with the 4 replicates samples for each animal clustering together. These delineations were most visible within the phyla level analyses. The clusters within the eukaryotic and bacterial populations, however, showed tighter clustering than was observed for the archaea population, probably because this phylum (archaea) makes up a very small proportion of the entire microbial community.

Comparisons between the results presented in this study and previously published investigations targeting DNA or RNA analysis in the rumen are difficult to make, as the methods of rumen sample preparation are very different. Anaerobic fungal sequences have been virtually absent from previous rumen metagenome and metatranscriptome studies (Hess et al., 2011; Ross et al., 2012; Dai et al., 2014; Kamke et al., 2016; Li et al., 2016; Comtet-Marre et al., 2017) indicating that methods of sample preparation have not been robust enough to isolate sequences from microbes growing deep within the plant fiber matrix of solids rumen samples. Differences in diet where concentrate levels are high could also preclude significant active anaerobic fungal contributions (Boots et al., 2013; Gruninger et al., 2014).

## Conclusion

This study set out to explore possibilities of reducing the quantity of materials and reagents that would need to be used to extract total RNA from solid-phase rumen digesta samples, as well as to develop a pipeline of analysis that would elucidate the total active microbial community structure. The purpose was to explore avenues for costs savings in projects involving RNA extraction and to develop a pipeline for total rRNA-Seq analysis. Our method successfully established the possibility of extracting RNA of high quality and integrity from an initial rumen solid digesta mass of as low as 2 g. We also successfully established that a mass of 0.6 g of the ground solid material can be successfully used in the actual RNA extraction procedure and that 5:1 TRIzol reagent/ground mass ratio is suitable for extraction of RNA of good quality rather than the current practice of a 10:1 ratio. This study further demonstrated the possibility of using RNA-Seq in phylogeny and taxonomy studies and in analysis pipelines as would be normally applied to traditional amplicon data by extracting appropriate regions of the total RNA information. This study is the first to elucidate simultaneously the relative active contributions of archaea, bacteria, protozoa and fungi to the rumen community. This method therefore combines the benefits of a breadth of coverage of RNA-Seq with the flexibility and specificity of a targeted amplicon approach, enabling more robust and appropriate statistical analysis of diversity beyond the mere elucidation of taxonomic profiles.

## Ethics Statement

All procedures and protocols used in this study were reviewed and approved by the Animal Care Committee at the Lethbridge Research and Development Centre of Agriculture and Agri-Food Canada. Care and management of heifers followed the guidelines of the Canadian Council on Animal Care.

## Author Contributions

CE and RF conceived the research. ZW, XW, and AR conducted the experiments. CE, ZW, and XW analyzed the data. CE, ZW, and RF wrote the paper. All authors approved the final manuscript.

## Conflict of Interest Statement

The authors declare that the research was conducted in the absence of any commercial or financial relationships that could be construed as a potential conflict of interest. The reviewer MP and handling Editor declared their shared affiliation, and the handling Editor states that the process nevertheless met the standards of a fair and objective review.
