# Supplementary material for: Total rRNA-Seq Analysis Gives Insight into Bacterial, Fungal, Protozoal and Archaeal Communities in the Rumen Using an Optimized RNA Isolation Method
Source: Front Microbiol. 2017 Sep 21;8:1814. doi: 10.3389/fmicb.2017.01814 (PMC5613150; doi:10.3389/fmicb.2017.01814)
Supplement: Supplementary file 1 [file Data_Sheet_1.PDF]

Table S.1: Breakdown of the sequence reads from total RNA analysis showing relative abundances of the phyla Archaea, Bacteria l, Protozoa and Fungi.

Treatment	Total reads (LSU$SSU)	Archaeal:16S_rRNA	Bacterial:16S_rRNA	Eukaryotic18S_rRNA	Total SSU	Total LSU	%Archaea	%Bacteria	%Eukaryote	(%Fungi	%Protozoa)
6_776-1	128492	777	28276	19096	48149	80314	1.61374068	58.7260379	39.660221	12.097269	27.562952
6_776-3	1086590	6376	247158	163482	417016	669404	1.528958122	59.2682295	39.202812	12.8920233	26.310789
6_778-1	910566	6142	188936	129556	324634	585495	1.891976811	58.1996957	39.908328	13.4402682	26.468059
6_778-3	797953	4706	173928	110243	288877	508624	1.629067042	60.208324	38.162609	11.4783794	26.68423
8_788-1	454367	2558	115889	49222	167669	286645	1.525624892	69.117726	29.356649	9.81819822	19.538451
8_788-3	33487	146	9564	3575	13285	20200	1.098983816	71.9909673	26.910049	8.18372058	18.726328
8_790-1	538204	3324	125342	59164	187830	350263	1.769685354	66.731619	31.498696	8.60737383	22.891322
8_790-3	273713	1347	63427	33343	98117	175538	1.37285078	64.6442513	33.982898	8.99627969	24.986618
10_800-2	824578	5544	176385	108606	290535	533962	1.908203831	60.7104135	37.381383	14.8630655	22.518317
10_800-3	410294	2847	87691	51629	142167	268043	2.002574437	61.6816842	36.315741	14.260958	22.054783
10_801-2	381665	2662	83650	52066	138378	243243	1.923716198	60.4503606	37.625923	13.0307121	24.595211
10_801-3	181024	1194	37056	23954	62204	118810	1.919490708	59.5717317	38.508778	12.8869286	25.621849
11_806-1	104098	571	24220	14812	39603	64480	1.441809964	61.1569831	37.401207	9.69118226	27.710025
11_806-2	650399	3557	143703	83663	230923	419389	1.540340287	62.2298342	36.229826	9.4632794	26.766546
11_808-2	727029	4449	159876	86609	250934	475310	1.772976161	63.7123706	34.514653	9.22768757	25.286966
11_808-3	274469	1789	68629	33741	104159	170209	1.717566413	65.8886894	32.393744	8.70017742	23.693567
14_818-1	578522	3011	105341	104978	213330	364993	1.411428304	49.3793653	49.209206	10.8725458	38.336661
14_818-2	416185	1830	75570	80813	158213	257895	1.156668542	47.7647222	51.078609	10.3655061	40.713103
14_820-1	39164	219	7440	7280	14939	24220	1.465961577	49.8025303	48.731508	8.03840518	40.693103
14_820-3	363376	1911	86291	77305	165507	197846	1.154633943	52.1373718	46.707994	8.81684554	37.891149
15_824-1	451476	3147	112903	48490	164540	286552	1.912604838	68.6173575	29.470038	8.24766853	21.222369
15_824-2	387064	2613	95846	41081	139540	246999	1.872581339	68.6871148	29.440304	8.25050887	21.189795
15_826-1	1063172	6334	241523	141224	389081	673745	1.627938655	62.0752491	36.296812	9.56357313	26.733239
15_826-3	202892	1192	47152	31045	79389	123396	1.501467458	59.3936188	39.104914	9.84326297	29.261651
**TOTAL**	**11278779**	**68246**	**2505796**	**1554977**	**4129019**	**7145575**					
Average	469949.125±62638.8	2843.6±390.5			172042±22787		1.61±0.05	60.92±1.29	37.46±1.31	10.48±0.43	26.97±1.28
Proportion (%)					37.01±0.48	62.95±0.46					

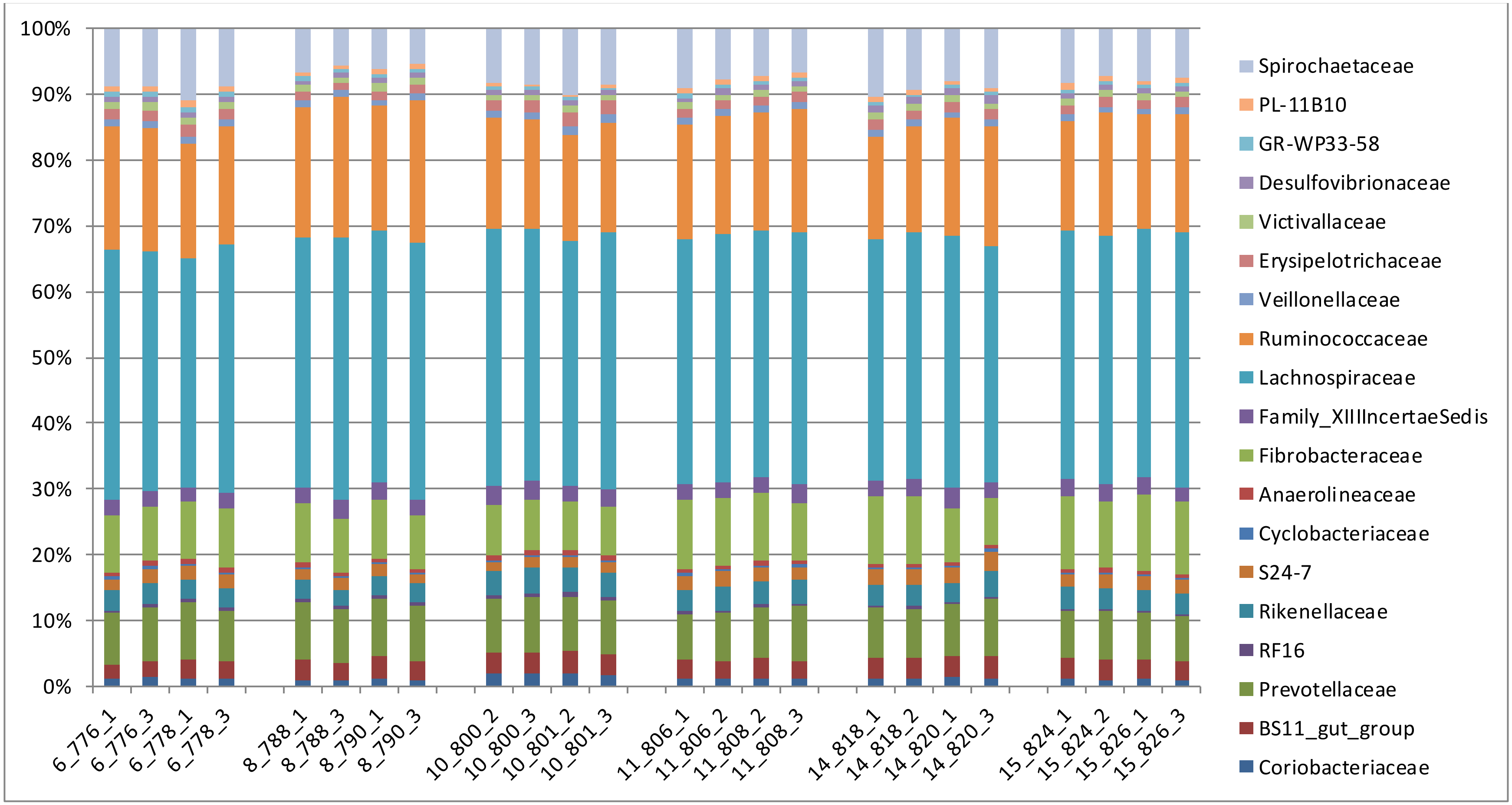

Figure S.1: Family level taxonomy profile for the bacterial phylum, showing relative abundances of specific families – obtained from total RNA

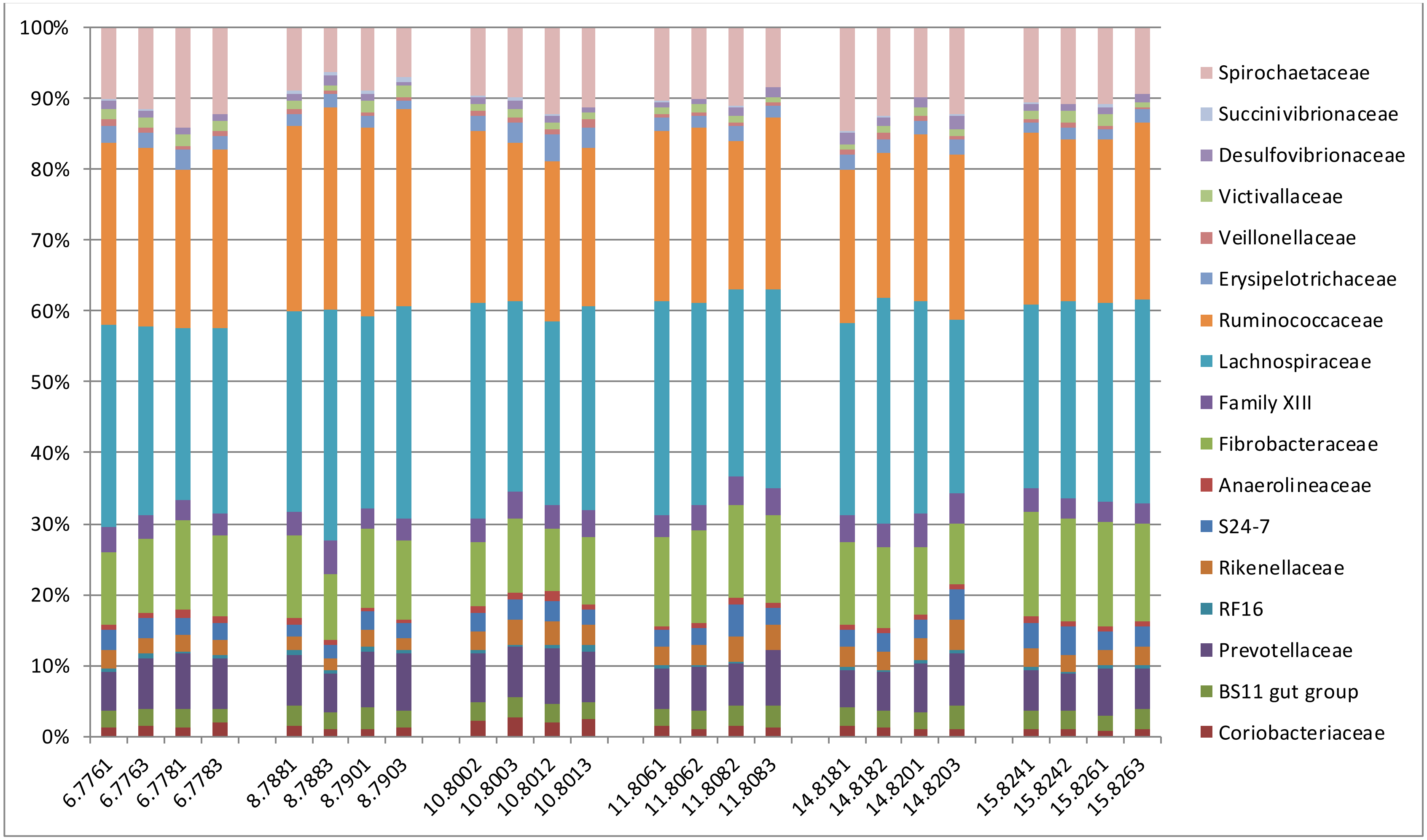

Figure S.2 Family level taxonomy profile for the bacterial community in the V4 region – showing relative abundances of the taxonomic classifications that were presented in figure S.1 (from total RNA data).

Table S.2 Summary of the reads extracted from the V4 region – for all the animals sampled.

Number of sequence reads in each total RNA sequence file analyzed: 200,000

Total no of reads in V4 region: 104467 - representing 2.17 % of the entire total RNA sequences analysed.

Number of samples: 24

Number of observations: 33902

Table density (fraction of non-zero values): 0.081

Counts/sample summary:

Min: 3078.0

Max: 9110.0

Median: 4146.000

Mean: 4352.792

Std. dev.: 1149.763

Observation Metadata Categories: taxonomy

Counts/sample detail:

11_8082: 3078.0	15_8241: 3277.0	8_7901: 3518.0	6_7783: 3727.0	10_8003: 3807.0	14_8181: 4281.0
11_8062: 3968.0	15_8242: 3349.0	8_7903: 3860.0	6_7781: 4182.0	10_8013: 3998.0	14_8182: 4708.0
11_8061: 4250.0	15_8261: 3895.0	8_7881: 4032.0	6_7761: 4204.0	10_8002: 4110.0	14_8201: 4907.0
11_8083: 4477.0	15_8263: 5557.0	8_7883: 4537.0	6_7763: 5365.0	10_8012: 4270.0	14_8203: 9110.0

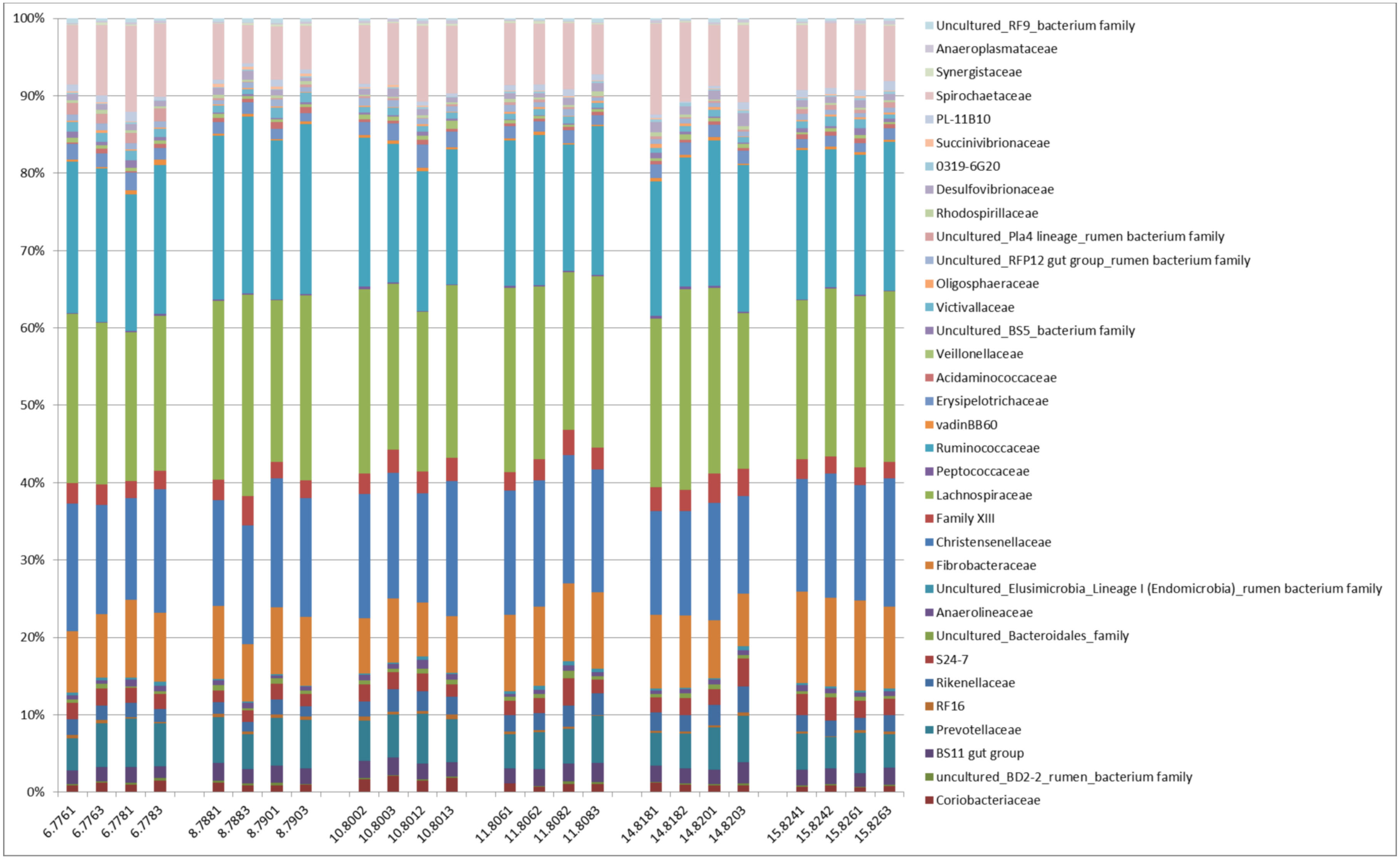

Figure S. 3: Family level taxonomy profile for the bacterial community in the V4 region - showing relative abundances for all families occurring in > 0.1% of the bacteria population

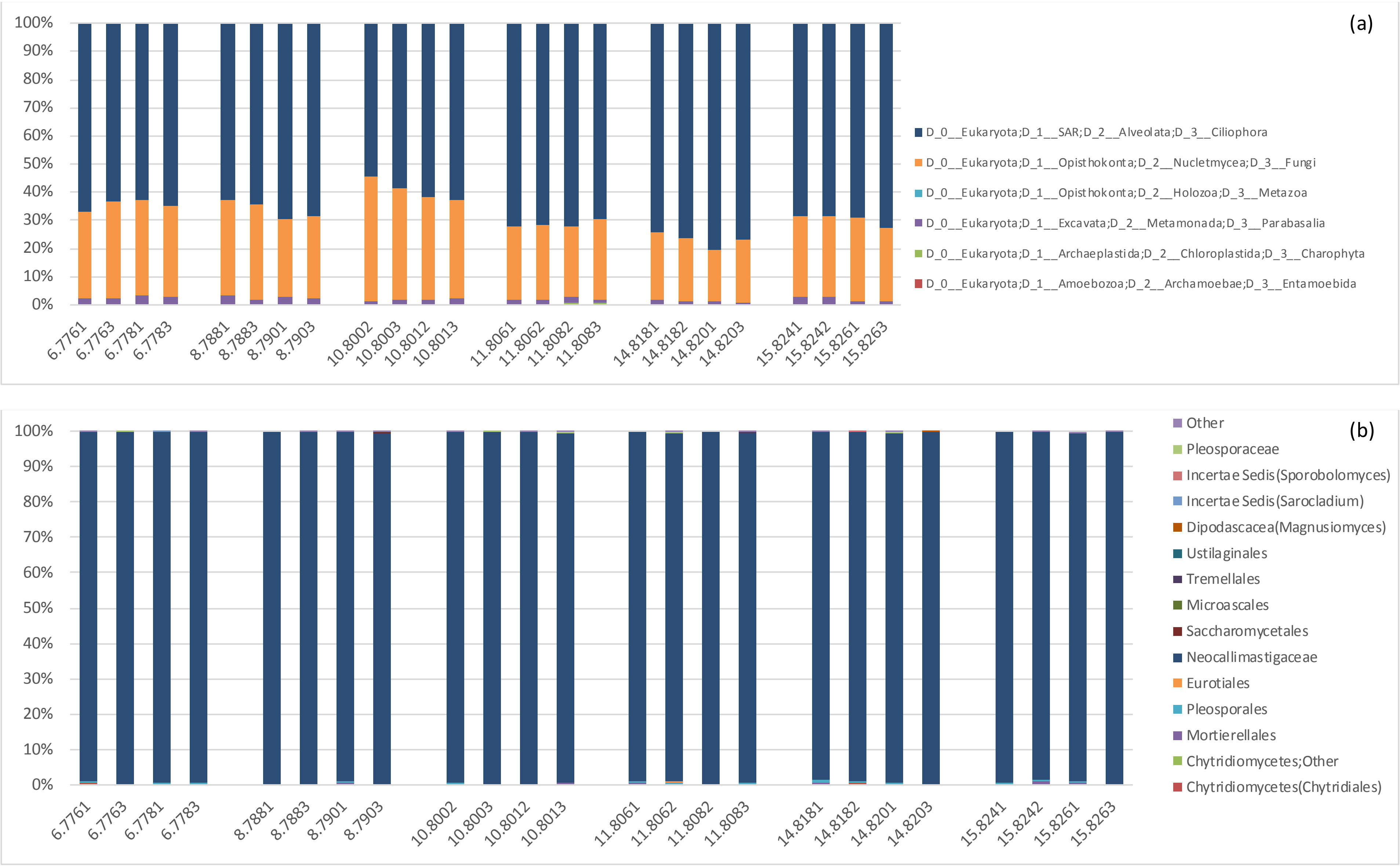

Figure S. 4: Relative abundances of the Eukaryotic community profiled from reads mapped to the V4 region of the rRNA gene. (a) the complete profile showing protozoans and fungi, and (b) profile of the fungi community extracted from (a) and showing the relative contributions of the various fungi families
